# A Novel Animal Model of Impaired Glucose Tolerance Induced by the Interaction of Vitamin E Deficiency and ^60^Co Radiation

**DOI:** 10.1155/2015/457246

**Published:** 2015-04-14

**Authors:** Yue Guan, Yan Cheng, Ying Yin, Jialin Duan, Guo Wei, Yan Weng, Chao Guo, Yanrong Zhu, Yanhua Wang, Miaomiao Xi, Aidong Wen

**Affiliations:** ^1^Department of Pharmacy, Xijing Hospital, Fourth Military Medical University, Xi'an 710032, China; ^2^Outpatient Department, Xijing Hospital, Fourth Military Medical University, Xi'an 710032, China

## Abstract

Impaired glucose tolerance (IGT), known as the prediabetes stage, is usually induced by habits of life or environmental factors. Established IGT animal models are mostly conducted with chemical compounds such as streptozocin or genetic modification. However, the occasion of exposure to these factors in daily life is seldom. The objective of this study was to establish a new animal model of IGT induced by VE deficiency in diet and exposure to radiation. SD rats were treated individually or in combination of these two factors. In the combination group, the calculated insulin sensitivity index decreased; then HOMA-*β* value increased. Oxidative damage and IGT were observed. Insulin secretion level in perfusate from pancreas response to glucose was characterized by a rapid but reduced first phase and an obviously defective second phase upon pancreas perfusion. Histopathological images demonstrated the pathological changes. Western blotting analysis showed that the insulin signaling pathway was downregulated. The interaction of VE deficiency in diet and exposure to radiation could break the equilibrium of oxidation and antioxidation and result in IGT. More importantly, a new IGT model was successfully established which may be conducive to further research into development of drugs against human IGT.

## 1. Introduction

Prediabetes is known as a transition stage from normal blood glucose to the diagnosis of diabetes. Up to 70% individuals with prediabetes eventually develop diabetes during many years or a short period [1]. Hence, a great many clinic investigations are focused on prediabetes. Impaired glucose tolerance (IGT) is considered as the prediabetic phase, which means a high risk for developing type 2 diabetes mellitus (T2DM) and complications (including cardiovascular, neuropathic, and nephropathic lesions). It is estimated that approximately 5–10% individuals with IGT develop to T2DM per year [1] and the number of adult with IGT would globally reach 472 million by 2030 [2]. As a high risk factor, IGT stage also provides the only opportunity to reverse to healthy condition in prediabetes, so it is the optimal timing for pharmaceutical intervention, and a satisfactory animal model to mimic IGT in vivo is vital for therapeutic drug development.

Most of established animal models are induced by chemical compounds (such as streptozocin, STZ) or genetically modification [3–5]. However, in daily life, the occasion of exposure to these analogous chemical substances or induction of genetic modification is uncommon. STZ treated or genetically modified rats themselves are obviously abnormal animals and these modified genetic patterns are not the same as those of diabetic patients. Thus, the generalization of the results from those artificially defective animals is confined when it comes to humankind [6, 7].

Oxidative stress is considered as a unifying mechanism in the development of diabetic complications; simultaneously, chronic hyperglycemia deteriorates oxidative stress damage [8]. Accumulating studies have provided data linking oxidative stress to IGT. Increased oxidative stress is presented in subjects with IGT [9], and an increase in glucose concentrations can lead to tissue damage by increasing oxidative stress [10]. Vitamin E (VE) is one of the typical antioxidant vitamins in our diet, protecting cells and tissues from free radicals damage. Deficiency of VE in body, not surprisingly, could induce oxidative stress. And radiation also as an independent element induces reactive oxygen species release [11]. Radiation, a natural phenomenon which exists widely in the universe and human living space, may cause and accelerate oxidative stress [12]. It does damage to health by inducing a great deal of free radicals that result in peroxidation of the whole body [13]. Hence, it is assumed that VE deficiency and radiation could induce IGT. To compare and analyze the effects of those two factors on induction of IGT, the exposure of radiation and the VE deficiency diet were included in this experiment.

Our direct aim is not T2DM but IGT, because IGT itself is a severe abnormal status for human and, especially, an inevitable stage before T2DM [14–16]. Therefore, it is meaningful to explore the risk factors leading to IGT so as to prevent its development. In the present study, since the rats treated with radiation and VE deprivation in diet together could suffer IGT, they can serve as a new animal model for further study.

## 2. Materials and Methods

### 2.1. Preparation of the Experimental Diet

Three kinds of feed were prepared in this study: purchased basal feed, high lipid and high sucrose feed (HH feed), and VE deficiency feed. The HH feed was made from the basal feed and some additional ingredients (Table 1). To obtain the VE deficiency feed, VE in the basal feed was deprived first by ultraviolet radiation. The single-layer basal feed were placed under short wave ultraviolet with 3 W/m^2^ radiation intensity for 5 h. They were mixed and spread again every 30 min. The height of ultraviolet light was 3 m.

Vegetable oil was added to dehydrated alcohol and the lower layer was collected and then heated 7 h at 220°C to remove the remaining alcohol and VE. The residual was exposed to ultraviolet light with a wavelength of 280~400 nm for 2 h. HPLC was employed to detect the existence of VE in the vegetable oil. VE standard solution was prepared by dissolving an accurately weighted standard of the compound in methanol to give the final concentration of 1 mg/mL and then stored at 4°C. HPLC conditions were as follows: chromatogram column was Hypersil C_18_ (5 *μ*m, 250 mm × 4.6 mm, Thermo); mobile phase was methanol: double-distilled water (99 : 1); column temperature was 25°C; flow rate was 1 mL/min; detection wavelength was 275 nm; and injection volume was 20 *μ*L.

VE deprived vegetable oil was mixed with the basal feed in the proportion as shown in Table 1. When consuming a high-fat diet, individuals develop increased plasma insulin levels, which eventually lead to the inability to maintain glucose homeostasis [17].

### 2.2. Source of Radiation

*γ* ray was chosen for its powerful penetrability. Steady state *γ*-radiolysis was carried out using ^60^Co *γ*-source with a dose rate of 4 Gy/min measured by standard Fricke dosimetry because of its mildness for rats.

### 2.3. Animals and Treatments

This study was approved by the Fourth Military Medical University Committee on Animal Care (Number fmmu-11-5201, approved on May 10, 2012). Fifty SD rats (male, weighing 190–220 g) were obtained from the Experimental Animal Research Center of the university and randomly divided into 5 groups, with 10 in each group. The basal diet was fed to the rats in the blank control group, and the HH diet was fed to those in the experimental control group. The rats in the radiation group got both HH feed and *γ*-radiation. VE deficiency feed was given to the rats in the VE deficiency group, and the rats in the VE deficiency/radiation group were treated with VE deficiency feed and simultaneous *γ*-radiation. During the experimental period, body weight and food intake of all rats were monitored weekly.

The rats are all fed with basal feed or HH diet or VE deficiency diet. Three times radiation treatments are implemented at 15th, 30th, and 60th day as shown in the upper dotted line frame, respectively. Different indexes detections in different days are carried out following the method displayed in the bottom dotted line frame.

The rats in the radiation group and the VE deficiency/radiation group received total-body *γ*-radiation at a dosage of 4 Gy on 15th, 30th, and 60th days, respectively. On 21th, 36th, and 66th days (the sixth day following the radiation), blood sugar, serum SOD activity, TBARS concentration, and AGEs fluorescence were detected. On 60th day, fasting serum insulin levels were measured; after that, fasting blood sugar was detected every 10 days. On 90th day, oxidative damage indexes were analyzed and intravenous glucose tolerance test (IVGTT) was conducted.

### 2.4. Analysis of Oxidative Damage Indexes

Superoxide dismutase (SOD), malondialdehyde (MDA), and advanced glycation end products (AGEs) were measured as the indexes for oxidative damage. Blood samples were collected from the tail veins of conscious nonfasting animals, and serums were separated by centrifuging for 10 min (5000 rpm/min). Levels of thiobarbituric acid reactive substances (TBARS) were determined as described [18]. The TBARS were calculated as MDA equivalents. SOD levels were measured according to the procedure reported in the literature [19]. Fluorometric value of AGEs was measured as previously described [20]. Fluorescence spectrophotometer was employed to detect the intensity.

### 2.5. Detection of Blood Sugar and Serum Insulin

The rats fasted overnight, and blood samples were taken from the tail veins of conscious animals. Blood glucose meter was employed to detect the blood sugar and radioimmunoassay was applied to detect serum insulin. Insulin sensitivity index (ISI) and homeostasis model assessment-*β* (HOMA-*β*) were calculated according to the following formulas [21]:

(1) $$\begin{array}{c} \text{ISI}=\ln\left(\frac{1}{\text{fasting}\;\text{blood}\;\text{glucose}\times \text{fasting}\;\text{serum}\;\text{insulin}}\right)\\ \text{HOMA-}\beta \;\text{value}=\frac{20\times \text{fasting}\;\text{serum}\;\text{insulin}}{\left(\text{fasting}\;\text{blood}\;\text{glucose}-3.5\right)}. \end{array}$$

The criterion for insulin resistance is set where there is a significant difference (*P* < 0.05) in the ISI and HOMA-*β* values between treatment groups and control.

### 2.6. Intravenous Glucose Tolerance Test (IVGTT)

To investigate IGT, IVGTT [7] was conducted on 90th day. In brief, after 12 h of fasting, glucose solution was injected into the lateral tail veins of conscious animals. Following successful infusion blood glucose was measured, respectively, before and 5, 15, 30, 120, and 180 min after the injection.

### 2.7. Pancreas Perfusions

The pancreas was perfused following a modification of a previously described method [22]. The rats were anesthetized with 1% pentobarbital sodium (60 mg/kg, i.p.). The whole pancreata were perfused continuously in situ with 2 mL/min Krebs-Ringer bicarbonate buffer, containing 2 g/L bovine serum albumin and 5 mmol/L glucose, bubbled with a mixture of 95% O_2_ and 5% CO_2_. After a 30 min equilibration period, the samples were collected, respectively, at 30 min, 40 min, and 45 min from the beginning of the perfusion. Then, glucose concentration in the perfusate was increased from 5 to 11 mmol/L and decreased back to 5 mmol/L at 65 min. The outflows were collected every minute during the 46 min to 50 min period and then every 5 min during the 55 min to 65 min period. The last samples were taken at 70 min and 80 min, respectively. The insulin concentrations in these samples were measured by radioimmunoassay.

**Figure 1:**
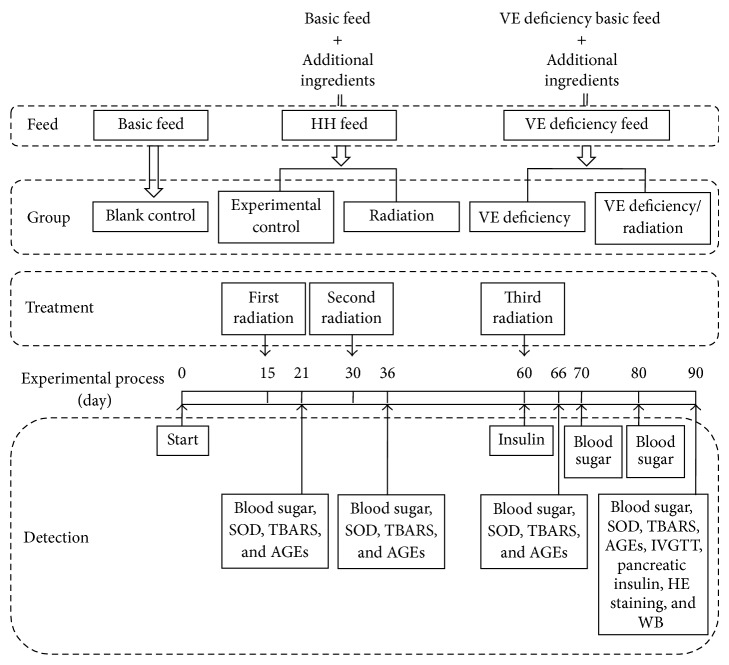
The schematic of the experimental process. The main timeline in the middle of this schematic represents the number of days. Rats are divided into 5 groups and fed with basal feed or HH feed or VE deficiency feed. Three times radiation treatments implement at 15th, 30th, and 60th day, respectively. Different indexes detections in different days are carried out following the method displayed in the bottom dotted line frame.

The experimental procedure of this study is shown in Figure 1.

### 2.8. Histopathological Examination

The excised pancreas tissues were immediately rinsed with normal saline and fixed in 10% neutral-buffered formalin solution. After embedding in paraffin, they were cut into 4 *μ*m thick sections. The sections were stained with hematoxylin-eosin staining (HE) for histopathological examination.

### 2.9. Western Blotting Analysis

Quadriceps muscle lysates were separated by 10% SDS-PAGE gel electrophoresis and transferred onto a nitrocellulose membrane and then blocked in 5% milk. Membranes were, respectively, incubated with antibodies at 4°C overnight. Primary antibodies against phosphor-insulin receptor substrate 1 (p-IRS1, Cat. number 2386), IRS1 (Cat. number 3407), phosphor-phosphoinositide-dependent protein kinase 1 (p-PDK1, Cat. number 3438), PDK1 (Cat. number 5662), phosphor-amino kinase terminal (p-Akt, Cat. number 4060) and Akt (Cat. number 4691) were obtained from Cell Signaling Technology. Antiglucose transporter 4 (GLUT4, Cat. number G4173) and GAPDH (Cat. number G9545) were obtained from Sigma. After being washed three times, membranes were incubated with HRP-conjugated goat anti-rabbit IgG (Bioss, Cat. number 140124) for 30 min at 37°C. The signals were detected using a chemiluminescent system, and the densitometry quantification was performed using an image analyzer Quantity One System. All protein quantifications were adjusted for the corresponding GAPDH level.

### 2.10. Data Analysis

Data were expressed as mean ± standard deviation (SD). The area under the curve for the blood sugar was calculated with the linear trapezoid method. Statistical analysis was performed by analysis of variance (ANOVA) followed by SNK-q test for multiple comparisons. Data analyses were performed using the SPSS 21.0 software. Differences were considered significant at *P* < 0.05.

## 3. Results

### 3.1. Chromatogram Qualitative Analysis

**Figure 2:**
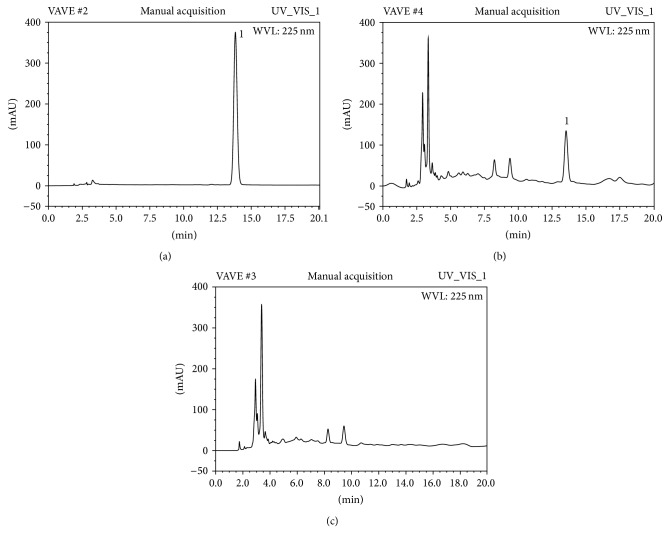
Representative HPLC chromatograms of VE standard substance (a), VE in the vegetable oil before the treatment (b), VE in the vegetable oil after the treatment (c); 1: chromatogram peak of VE. After the treatment, VE was almost all removed from the vegetable oil.

To investigate whether VE in feed was deprived thoroughly, qualitative analysis was performed using HPLC. Chromatograms of VE standard substance, VE in the vegetable oil before and after treatment are shown in Figure 2. As indicated in the chromatograms, impurities in the vegetable oil did not interfere with the detection of VE under the given HPLC conditions. Accordingly, when extracted and boiled, VE in the vegetable oil was destroyed.

### 3.2. Body Weight and Food Intake

**Figure 3:**
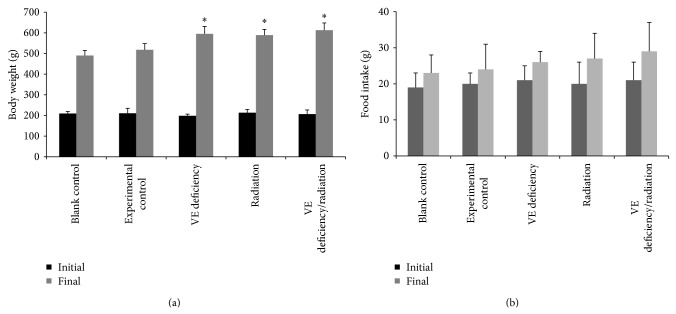
Effect of VE deficiency and/or radiation on body weight (a) and food intake (b) in rats. The values are expressed as mean ± SD (*n* = 10). ^*^ *P* < 0.05 as compared with experimental control group.

Body weight of rats and food intake were recorded every week.  Figure 3 indicated the changes of body weight and food intake on the initial and the final day of the experiment. There was no significant intragroup variation in the basal body weight and food intake of all rats before the experiment. After being induced by HH diet, the rats in experimental group exhibited a slight increase of body weight. Treatment with VE deficiency diet and/or radiation improved the body weight and food intake. These results demonstrated that the rats got the early syndrome of diabetes.

### 3.3. Oxidative Damage Indexes

**Figure 4:**
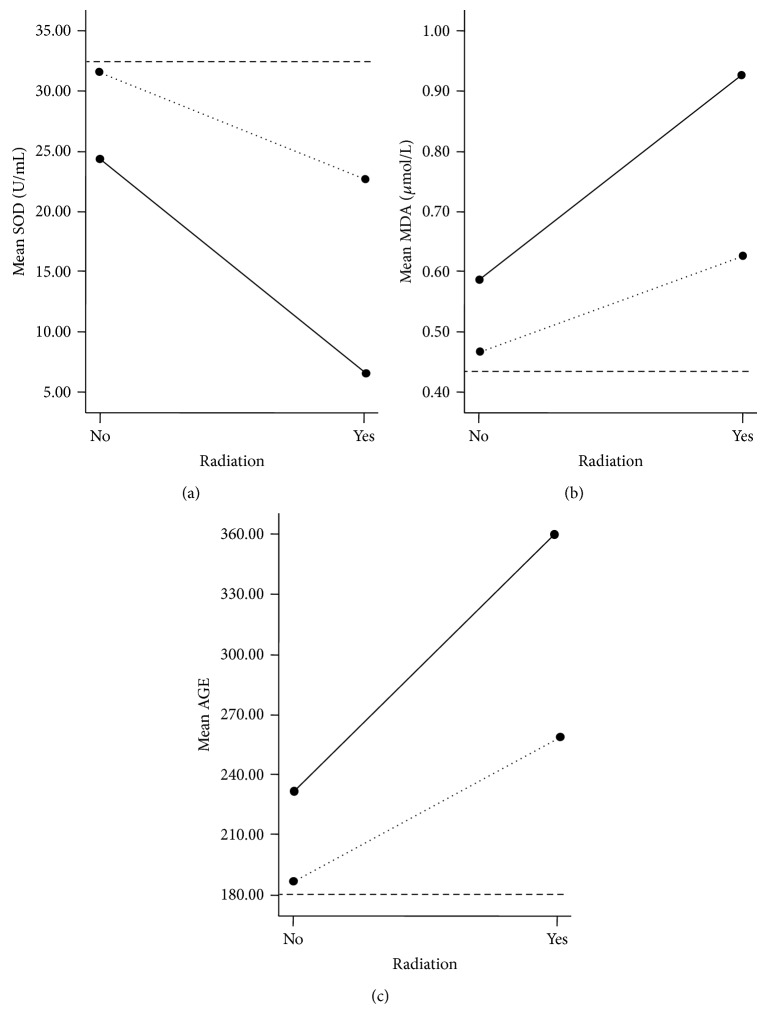
The interaction of VE deficiency and radiation measured by the mean of SOD, MDA, and AGE, respectively, on 66th day. The black dots indicate the mean of the corresponding index. The solid lines represent VE deficiency. The dotted lines represent radiation, and the discontinuous lines represent experimental control. As shown in (a), the dotted line is downward from radiation “no” to “yes,” which indicates radiation decreases the SOD activity comparing to the experimental control. The solid line slopes more than that of the dotted line, which means VE deficiency makes radiation more seriously lower the SOD activity. The similar analysis is worth applying to (b) and (c). With the two unparalleled lines (the solid and the dotted) in each graph, it is shown that the effect differences in VE deficiency depend on the presence of radiation, which gives a clue to the existence of interaction between VE deficiency and radiation and implies that the combination of these two factors could cause a magnified effect of oxidative stress.

The effects of oxidative stress were checked at different stages of the experiment as previously described, and no significant differences were found between the four experimental groups and the blank control group until 66th day. As shown in Table 2, on 66th day, except those in the experimental control group, the rats in three experimental groups all showed marked oxidant damage, as compared with those in the blank control group. Notably, the maximum damage was observed in the VE deficiency/radiation group, which might not be considered just as a result of a combined effect from the two factors because the SOD activity, MDA level, and AGE in the group showed significant difference from those in the other two treated groups (*P* < 0.05). As shown in Figure 4, the two lines that represent the two types of status of VE deficiency appeared unparallel, and the gap between them became bigger when radiation existed, which may imply an obvious magnification of the effect. The interaction between the two factors was confirmed in the end by two-way ANOVA (*P* < 0.05).

### 3.4. Blood Sugar and Serum Insulin

The levels of blood sugar and serum insulin were checked at different stages of the experiment as previously described. On 60th day, all rats developed insulin resistance except rats in the blank control group and the experimental control group. The VE deficiency/radiation group rats were observed with a level of fasting serum insulin and HOMA-*β* value significantly higher than those in other four group rats (*P* < 0.05) but with a distinctive decrease in insulin sensitivity index (*P* < 0.05) (Table 3).

### 3.5. Induction to Intravenous Glucose Tolerance Test (IVGTT)

**Figure 5:**
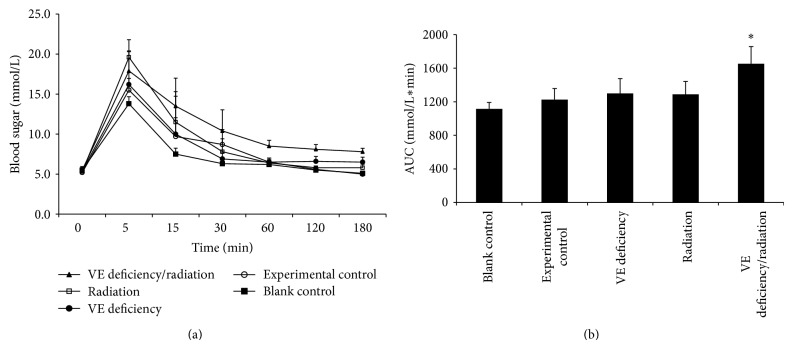
Time course of blood sugar on 90th day during IVGTT. (a) Blood glucose curve following IVGTT. Data are presented as means of ten observations in each group. (b) IVGTT area under the curve from 0 to 180 min. ^*^ *P* < 0.05 as compared with the experimental control group.

The findings of IVGTT conducted on 90th day are shown in Figure 5(a). During the procedure, the blood glucose level reached the steady state level between 30 and 180 min after the caudal vein injection of glucose. The blood sugar level of the blank control group returned to basal value within 30 min. In the VE deficiency/radiation group, an obvious IGT was observed, that is, blood sugar level attained 17.9 ± 5.4 mmol/L at 5 min following the injection, and decreased slowly to 7.8 ± 0.4 mmol/L at 180 min, significantly higher than that of the other four groups (*P* < 0.05). There was no significant difference in blood sugar level between the experimental control group and the blank control group (*P* > 0.05). The AUC measures were determined from 0 to 180 min (Figure 5(b)). The VE deficiency/radiation group had significantly higher AUC-IVGTT values in comparison to the experimental control (*P* < 0.05).

### 3.6. Insulin Secretion Response to Glucose from Pancreas

**Figure 6:**
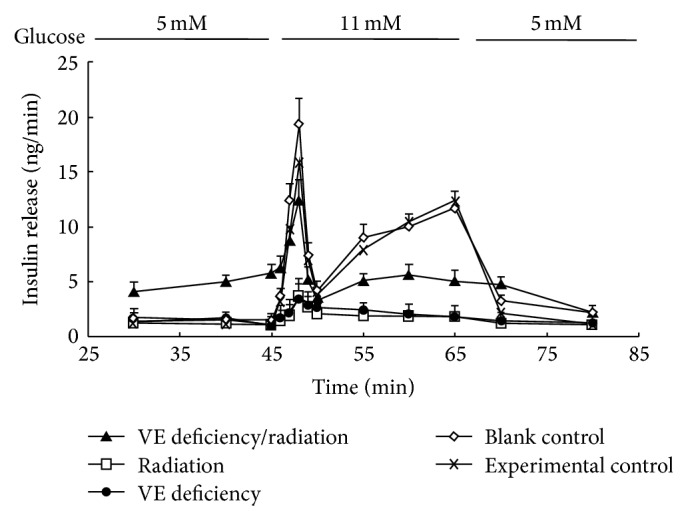
Insulin release from the pancreas of the different treated rats. Pancreas were perfused with a solution containing 2 g/L bovine serum albumin and 5 mmol/L glucose at the flow rate of 2 mL/min after a 30-min equilibration period. Glucose concentration was changed to 11 mmol/L at 45 min and then changed back to 5 mmol/L at 65 min. Perfusate samples were collected at 30, 40, 45, 46, 47, 48, 49, 50, 55, 60, 65, 70, and 80 min from perfusion beginning, respectively. Data are presented as mean ± SE of ten observations in each group.

The secretion of insulin from the pancreas was stimulated by increasing glucose concentration from 5 mmol/L to 11 mmol/L. The tendency of insulin releasing in each group is displayed in Figure 6. From the early stage of the perfusion till glucose concentration change, the secreted insulin levels of rats in the VE deficiency/radiation group were much higher than those in the blank control group. In the first phase of glucose-stimulation, the insulin release peak values of the four treatment groups, the experimental group, the VE deficiency group, the radiation group, and the VE deficiency/radiation group, were 97%, 18%, 20%, and 64% of blank control group, respectively, while, in the second phase, the insulin sensitivity decreased sharply, resulting in the AUC of 30–80 min period being 79 ± 2 ng and 86 ± 6 ng of the VE deficiency group and the radiation group, respectively, and showing the significant differences as against that of the blank control group of 267 ± 9 ng (*P* < 0.05). Interestingly, the situation in the VE deficiency/radiation group displayed a little difference. During the 45 min to 70 min period, its insulin concentration was higher than that of the other two treatment groups but still significantly lower than that of the blank control group (*P* < 0.05). Besides, there was no significance in AUC between the blank control and the experimental control group.

### 3.7. Histological Changes

**Figure 7:**
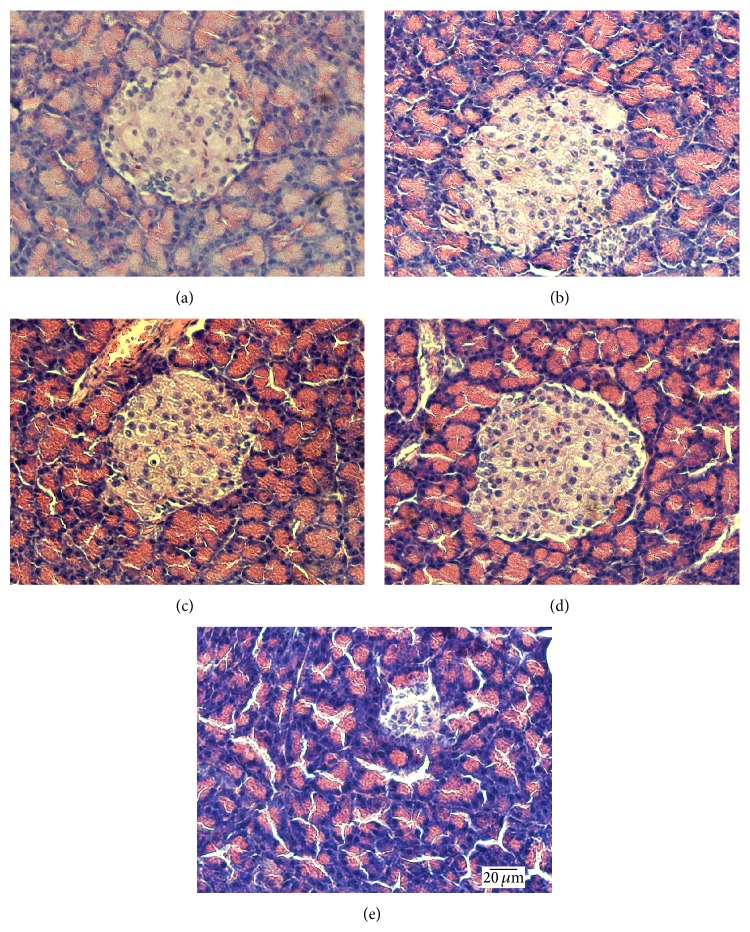
Histological changes of pancreatic islet tissue (HE × 400). (a)–(e) Blank control, experimental control, radiation, VE deficiency, and VE deficiency/radiation.

The results of histopathologic examination of pancreas in rats of all groups are shown in Figure 7, which supported all the findings above.  Figure 7(a) shows the normal appearance of islet cells in the pancreas, while, in Figures 7(b), 7(c), and 7(d), the islets were slightly damaged with the mass of the viable cell a little decreased and vacuolation detected. VE deficiency and radiation combined to aggravate the damage of islets in the pancreas (Figure 7(e)). The number and size of pancreatic islets were significantly decreased, and vacuolation and invasion of connective tissues were even serious.

### 3.8. Insulin Signaling Protein Analysis

**Figure 8:**
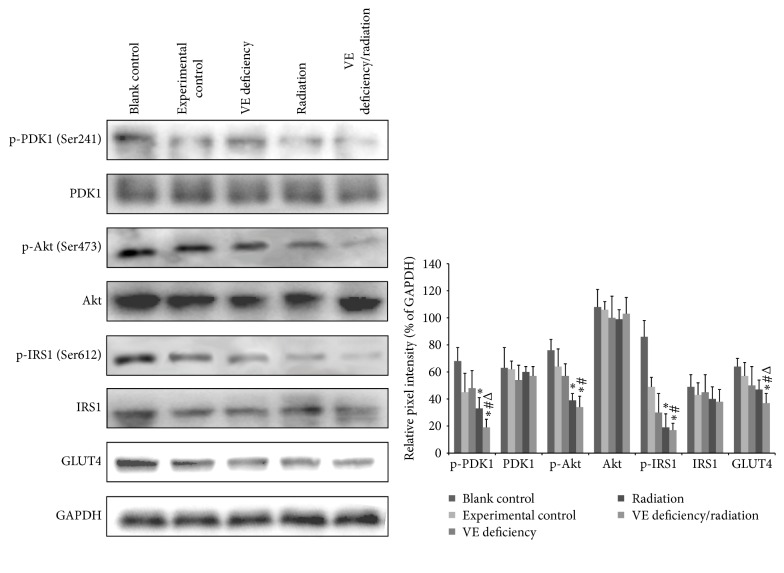
Expression of insulin signaling proteins following different treatments. Western blotting was performed to assess the expression of p-PDK1, PDK1, p-Akt, Akt, p-IRS1, IRS1, and GLUT4. The values from densitometry were normalized to the level of GAPDH, respectively. ^*^ *P* < 0.05 compared to the corresponding experimental control group, ^#^ *P* < 0.05 compared to the corresponding VE deficiency group, and ^Δ^ *P* < 0.05 compared to the corresponding radiation group.

In order to confirm the activation of insulin signaling pathway, key proteins were detected using western blotting. As shown in Figure 8, treatment with VE deficiency diet or radiation downregulated the expression levels of p-PDK1 and p-Akt. Moreover, the VE deficiency/radiation group rats showed more downregulation of p-PDK1, p-Akt, p-IRS1, and GLUT4. In the meantime, the expression of PDK1, Akt, and IRS1 of all groups showed no significant difference.

## 4. Discussion

Although studies have found the evidence for the development of IGT into T2DM under the influence of oxidative stress, it remains unknown whether oxidative stress caused by external factors may induce IGT in healthy individuals. That is what we intended to investigate into. In addition, we lay our focus on IGT rather than on T2DM itself because we think it might be of great significance from the viewpoint of disease prevention. IGT as prediabetes was vital for therapeutic strategy. It would be very meaningful to set up an appropriate animal model for treatment exploration. Regretfully, the up-to-now established animal model was induced by the chemical compound we seldom contacted normally. We chose two daily contactable oxidative stress factors, antioxidant VE and radiation, as influencing factors to induce IGT in rats.

In clinic, patients with IGT got impaired in blood sugar value and insulin secretion. During this phase, the syndrome of diabetes may not show up yet. In our study, one or two factors treatment significantly increased body weight. This is similar to the clinical manifestation of diabetic patients in early stage as they are hyperphagic and gaining body weight. To the best of our knowledge, no interaction study on induction IGT between VE deprivation and exposure to radiation has been reported yet. Basic feed contains various essential antioxidants for bodies; depletion of antioxidants (such as VE) results in metabolism disorders, and aggravation of free radicals chain reaction, oxidation and peroxidation injury of RNA, DNA, protein, enzyme and biomembrane, and final induction of diseases [23]. Our results may suggest that all rats treated with VE deficiency in diet or radiation would be in disequilibrium of oxidation and antioxidation, and the even worse situation could exist under the interaction between VE deficiency and radiation exposure.

During the experiment process, the most serious abnormalities were observed in the rats fed on HH diet deficient VE and exposed to radiation; the oxidative damage indexes (SOD, MDA, and AGEs) of this group changed significantly on 66th day compared with those of other groups. While insulin resistance occurred in the three treated groups on 60th day. HOMA-*β* is frequently used to evaluate the function of islet beta cells of individual indicators. The group treated with both factors showed the lowest ISI, the highest HOMA-*β* value, and the most extreme oxidative damage indexes, implying oxidative stress could accelerate the development of insulin resistance. On the 90th day, IGT, as expectedly, occurred due to the most severe oxidative damage among the rats treated with both factors. Through all these phenomena, there existed an explicit chain that some risk factors of oxidative damage were imposed on rats first, then oxidative stress was observed followed by insulin resistance, and finally IGT developed. Based on all evidences, we would like to infer that oxidative stress should be a cause of IGT.

Another intriguing finding in our study was that when VE deficiency and radiation cooperated, their effects could interact. The synergistic action could be found in the data from rats treated with both factors and the interaction was confirmed by the statistical test performed. VE, one of the crucial antioxidants in organism, plays an important part in capturing and cleaning up excess free radicals as well as maintaining the balance of oxidation and antioxidation; thus lack of it will reduce body's antioxidative ability [24, 25]. Radiation damage produces large numbers of active oxygen free radicals in body and then causes chain reaction of free radicals [26]. Therefore, under the condition of severe VE deficiency, radiation will deteriorate organism's status of oxidative stress without the defense from VE. The severe oxidative stress then intensifies insulin resistance and the continual declining of insulin sensitivity will lead to irritable increment of insulin. From the histopathologic examination of pancreas, we can also see the similar changes, which assume to the damage of pancreatic islet. Consequently, such consumptive process can greatly impair the normal function of insulin and finally lead to IGT.

In the attempt to observe insulin response to glucose, pancreas perfusion was tested based on in vitro functional studies. Indeed, the perfused pancreata of animals treated with VE deficiency diet and exposure to radiation responded to an elevation of glucose concentration from 5 to 11 mmol/L, showed a biphasic pattern of insulin secretion which was characterized by a rapid but reduced first phase and an obviously defective second phase. Compared with the reported results in GK model rats [6], insulin secretion level in our study was manifested to be abnormally higher than that of the blank control at the early stage of perfusion (glucose concentration at 5 mmol/L), which was probably because insulin resistance demands more insulin secretion from pancreatic *β* cells. Progressive deterioration of *β* cell function in response to a glucose challenge has been demonstrated in individuals with prediabetes [27]. In the present study, the insulin concentration under this treatment peaked at 64% of the blank control value, suggesting that *β* cells still performed the function of insulin secretion to a certain extent, which is similar to the clinical symptom of type 2 diabetes patients. The rats in the radiation group and the VE deficiency group seemed insensitive to glucose, which may be because of the general injury. However, the exact reason needs further exploration.

Physiologically, the insulin signaling pathway is activated by external stimulus to enhance insulin secretion. However, when IGT appears, insulin activity decreases though the secretion amount increases. In the VE deficiency/radiation group, though the insulin release was higher than that of monofactor treatment according to pancreatic perfusion results, the blood glucose did not drop more during IVGTT. Instead, the blood sugar was increased. This amount increased but activity decreased insulin failed to activate the pathway. These were consistent with the downregulation of insulin pathway. Therefore, the insulin signaling pathway downregulation is likely an important mechanism for IGT induced by the cooperation of VE deficiency in diet and exposure to radiation.

Several selective kinds of inbreeding of rodent animals that develop the T2DM-like phenotype have generated many of the strains used today, in attempt to gain insights into the human condition. Ob/ob mouse, db/db mouse, and fa/fa rat are monogenic models of obesity, which all target on leptin gene [28–32]. Goto-Kakizaki (GK) rat is one of the best characterized animal models of spontaneous T2DM. Initially, Wistar rats with glucose tolerance at upper limit were selected for inbreeding. The 30th generation rats exerted steady diabetes [33]. Research on the offspring demonstrated that the quantitative, morphological, and functional deficiencies of *β* pancreatic cell from GK rat were not influenced by environment but genetic background [34]. The dysfunctional* ide* allele was unique to the diabetic phenotype of GK rat [35]. KK mouse is another widely used model of diabetes and obesity rather than GK rats including the superiority in simulating human obesity and generally easier production (animal models of diabetes mellitus). Due to the fact that these models are all genetically modified, it is difficult to ensure entire genetic homogeneity among inbred strains.

STZ treatments are also usually applied as T2DM model. STZ-induced rats are found weight loss, whereas our established model shows weight increase. Because STZ is a toxin to *β* cells leading to a severe reduction in functional *β* cells mass, and finally a typical diabetic symptom [36, 37]. Nevertheless, our method did not damage too much. More importantly, in human, T2DM is always induced by circumstance or habits of life instead of chemical compound or genetic variances. It is reported that high-energy diet could induce a prediabetic state including impairing reproduction function by modulating overall testicular metabolism [38, 39]. In comparison, our established model is aimed at imitating the prediabetic situation induced by not only feed factor but also environmental radiation. The effects of external risk factors on IGT generation were our concerned, which had no reports before.

## 5. Conclusion

This study has proved that oxidative stress may be an incentive of IGT with the fact that the latter was induced by the interaction between VE deficiency in diet and exposure to radiation. The interaction between these two factors implies that a comprehensive protection against oxidative damage can be more effective than the measures that deal with one risk factor at a time. Furthermore, a new animal model of IGT treated with radiation and VE deprivation in diet is established which may well simulate clinical symptom of T2DM patients and may be of greater significance for progress of disease prevention.

## Figures and Tables

**Table 1:** Proportion of the ingredients in feed.

Ingredients	Proportion (%)
Basal feed or basal feed free of VE	62.5
Lard oil	10.0
Cholesterol	2.0
Yolk powder	5.0
Sodium cholate	0.5
Saccharose	20.0
Total	100.0

**Table 2:** Effects of oxidative stress of the treatment groups on 66th day ($\overset{-}{x}\pm s$, *n* = 10).

Treatment	Serum SOD activity (U/mL)	Serum MDA concentration (*μ*mol/L)	Serum AGE fluorometric value
Blank control	31.70 ± 8.28	0.465 ± 0.120	189.20 ± 22.48
Experimental control	29.96 ± 5.31	0.498 ± 0.105	194.21 ± 27.10
Radiation	22.77 ± 6.12^ab^	0.625 ± 0.133^ab^	256.75 ± 30.79^ab^
VE deficiency	24.27 ± 3.23^ab^	0.586 ± 0.142^ab^	234.52 ± 35.35^ab^
VE deficiency/radiation	6.77 ± 3.61^abcd^	0.927 ± 0.161^abcd^	358.03 ± 58.82^abcd^

^a^ *P* < 0.05 versus blank control, ^b^ *P* < 0.05 versus experimental control, ^c^ *P* < 0.05 versus VE deficiency, and ^d^ *P* < 0.05 versus radiation.

**Table 3:** Fasting blood sugar, serum insulin level, and ISI on 60th day ($\overset{-}{x}\pm s$, *n* = 10).

Treatment	Fasting blood sugar (mmol/L)	Insulin (mU/L)	ISI	HOMA-*β*
Blank control	4.7 ± 0.4	20.67 ± 7.29	−4.6 ± 0.3	344.5 ± 28.4
Experimental control	4.9 ± 0.4	25.54 ± 5.36	−4.8 ± 0.2	364.9 ± 30.1
Radiation	5.2 ± 0.6	33.17 ± 5.96^ab^	−5.1 ± 0.2^ab^	390.2 ± 21.9^ab^
VE deficiency	5.1 ± 0.6	28.71 ± 2.78^ab^	−5.0 ± 0.2^ab^	358.9 ± 25.3^ab^
VE deficiency/radiation	5.4 ± 0.7	43.74 ± 5.81^abcd^	−5.5 ± 0.2^abcd^	460.4 ± 31.7^abcd^

^a^ *P* < 0.05 versus blank control, ^b^ *P* < 0.05 versus experimental control, ^c^ *P* < 0.05 versus VE deficiency, and ^d^ *P* < 0.05 versus radiation.
